# Aufsuchende Behandlung an den Universitären Psychiatrischen Kliniken Basel: Vorstellung der Pilotprojekte Re-ACT und ACTTIV und Ergebnisse einer qualitativen Zuweisendenbefragung

**DOI:** 10.1055/a-2587-7093

**Published:** 2025-06-12

**Authors:** Katja Rother, Julian Möller, Franziska Rabenschlag, Mariela Jaffé, Christine Althaus Aebersold, Joerg Eysell, Constantin Bruttel, Lukas Imfeld, Andre Nienaber, Undine E. Lang, Christian Huber

**Affiliations:** 1 27250 Universitäre Psychiatrische Dienste BernBernSwitzerland; 2 27251 Universitäre Psychiatrische Kliniken BaselBaselSwitzerland; 3 27209 Abteilung Klinische Psychologie und Epidemiologie, Universität BaselBaselSwitzerland; 4 27188 Zentralinstitut für Seelische GesundheitMannheimGermany

**Keywords:** Aufsuchende Behandlung, intermediäre psychiatrische Versorgung, Zuweisungsprozesse, Assertive community treatment, intermediate psychiatric supply of treatment, referral processes

## Abstract

**Ziel:**

Die längerfristige Behandlung (Re-ACT) und Übergangsbehandlung (ACTTIV) sind zwei neu implementierte, aufsuchende Pilotprojekte der Universitären Psychiatrischen Kliniken Basel (UPK). Im Rahmen der Implementierung erfolgt eine Begleitevaluation, aus der erste Ergebnisse in Bezug auf den Zuweisungsprozess aus dem stationären Setting vorgestellt werden.

**Methodik:**

Um die Pilotprojekte erfolgreich zu etablieren wurden die Erfahrungen und Sichtweisen der zuweisenden Ärzt:innen in die aufsuchende Behandlung exploriert. Drei halbstrukturierte Fokusgruppeninterviews wurden durchgeführt.

**Ergebnisse:**

In der Inhaltsanalyse nach Mayring wurden als relevante Themen (1) das Anmeldeprozedere, (2) die Abgrenzung zu bestehenden Versorgungsangeboten in der Region, (3) die Patient:innenerfahrungen mit den Angeboten, (4) die Indikationsstellung und (5) die Erwartungen der Zuweisenden an das aufsuchende Angebot aufgenommen. Die Zuweisenden bewerteten die aufsuchenden Behandlungsangebote als hilfreich und wirksam. Sobald Patient:innen in Kontakt mit der aufsuchenden Behandlung gekommen sind, berichten sie gegenüber den Zuweisenden von positiven Erfahrungen. Gewünscht wurde eine zeitliche Flexibilisierung des Anmeldeprozederes, sodass eine Anmeldung auch nach Entlassung aus dem stationären Setting möglich ist. Weiterhin könnten mehr Informationen für eine systematisierte Indikationsstellung und Abgrenzung zu bestehenden Angeboten hilfreich sein.

**Schlussfolgerung:**

Die neu implementierten aufsuchenden Versorgungsangebote der UPK Basel werden auch von Seiten der Zuweisenden positiv bewertet. Diese Angebote können eine Behandlungslücke zwischen stationärer und ambulanter Versorgung schliessen und dabei auf die individuellen Bedürfnisse der Patient:innen eingehen. Während die Zuweisenden von positiven Erfahrungen berichten, ist weitere Forschung nötig, um besonders die Perspektive der Patient:innen systematisch zu verstehen.

## Abstract (en)

**Objective:**

The recovery oriented Assertive Community Treatment (Re-ACT) and the Assertive Community Treatment – Transitional Intervention (ACTTIV) are two newly implemented assertive outreach pilot projects of the University Psychiatric Clinics Basel (UPK). Initial results of evaluation conducted along with their implementation are presented here regarding the referral process from the inpatient setting.

**Methods:**

In order to successfully establish the pilot projects, experience and perspectives of referring physicians to the outreach treatment were explored. Three semi-structured focus group interviews were conducted.

**Results:**

Qualitative content analysis according to Mayring identified five topics: (1) registration procedure, (2) differentiation from existing care options in the region, (3) patients’ experiences with the services, (4) indication for treatment and (5) expectations of the referring physicians regarding the outreach treatment. Referring physicians rated the outreach treatment as helpful and effective. As soon as patients came into contact with the assertive community treatment, they reported positive experiences to the referring physicians. There was a desire for more flexible registration times so that admission would also be possible after discharge from the hospital. Furthermore, more information could be helpful for a systematised assessment of indications and for distinguishing the new treatment options from existing services.

**Conclusion:**

The newly implemented outreach services at UPK Basel are viewed positively by referring physicians. These services can close a treatment gap between inpatient and outpatient care and address the individual needs of patients. While referring physicians report positive experiences, further research is needed, in particular, to gain a better understanding of the patients’ perspectives.

## Einleitung

Die für den deutschsprachigen Raum entwickelte S3-Leitlinie „Psychosoziale Therapien bei schweren psychischen Erkrankungen“ 1 empfiehlt, dass „Menschen mit chronischen und schweren psychischen Störungen […] die Möglichkeit haben [sollten], auch über einen längeren Zeitraum und über akute Krankheitsphasen hinausgehend, nachgehend aufsuchend in ihrem gewohnten Lebensumfeld behandelt zu werden“ 1 26 . Eine aufsuchende Behandlung im gewohnten Lebensumfeld der Person kann durch das sogenannte Assertive Community Treatment (kurz ACT) abgebildet werden 2 . Ein multiprofessionelles Team bietet hierbei eine den individuellen Bedürfnissen der Person angepasste Behandlung an, die in der vertrauten Umgebung und im Zuhause der Personen stattfindet 3 , und damit den Empfehlungen aus den Leitlinien entspricht. Ein bedarfsdeckendes Versorgungsangebot liegt gegenwärtig in der Schweiz jedoch nicht vor 4 , was auch auf die sektorisierte Steuerung und Finanzierung der Gesundheitssysteme zurückzuführen ist 5 6 . Die einzelnen HT Versorgungsmodelle unterscheiden sich zudem zum Teil deutlich voneinander. Die grössten Unterschiede zeigen sich in den Einschlusskriterien der Patient:innen sowie in der Verfügbarkeit des multiprofessionellen Teams im Sinne der zeitlichen Behandlungsintensität 5 6 7 8 .

### Aufsuchende Behandlung in den UPK Basel

Aufsuchende Behandlung bzw. Assertive Community Treatment kann besonders nach einem stationären Aufenthalt in einer psychiatrischen Klinik relevant sein. Der Wechsel aus einem sehr strukturierten und eng betreuten stationären Setting kann abrupt und daher destabilisierend sein. Personen erhalten mit einem Mal weit weniger Unterstützung bei der Aufrechterhaltung ihrer Therapien. Etwaige ambulante Angebote vermögen dies nur bedingt abzufedern. Es fehlt ein holistisches intermediäres Angebot, das Personen dabei unterstützen kann, den Übergang in das vertraute Umfeld so zu gestalten, dass eine weitere Stabilisierung erfolgen kann, die eigenen Bedürfnisse wieder erfüllt und Ziele verfolgt werden können und die Wahrscheinlichkeit für potenzielle Rückfälle reduziert wird 9 10 .

Um diese Lücke zu schliessen wurde an den Universitären Psychiatrischen Kliniken Basel (UPK) im Kanton Basel-Stadt ein aufsuchendes Behandlungsangebot (ACT) entwickelt, das ihm Rahmen von zwei Pilotprojekten von 2019 bis 2021 implementiert und evaluiert wurde. Dabei handelt es sich um ein Programm für eine längerfristige Recovery-orientierte aufsuchende Behandlung für Personen mit einer hohen Inanspruchnahme von stationären psychiatrischen Behandlungen (Re-ACT; Recovery oriented Assertive Community Treatment) sowie um ein aufsuchendes Angebot zur Übergangsbehandlung (maximal 3 Monate) von Personen nach einem stationären Klinikaufenthalt (ACTTIV; Assertive Community Treatment – Transitional Intervention).

In den aufsuchenden Behandlungsprogrammen der UPK Basel erfolgt die Triagierung und Zuweisung der Patient:innen primär durch die Behandler:innen auf den jeweiligen Stationen (im Folgenden als Zuweisende bezeichnet). Die Zuweisenden fungieren also als Schnittstelle zwischen dem stationären Aufenthalt und der ambulanten Behandlung. Sie können dabei Erwartungen wecken und Vorabinformationen teilen. Wenn dieser erste Kontakt erfolgreich gelingt, kann dies zur Behandlungsteilnahme und auch zur Behandlungszufriedenheit und -qualität beitragen. Bisher liegen jedoch nur wenige Studien vor, die den Prozess der Zuweisung bei aufsuchenden psychiatrischen Behandlungsangeboten untersuchen. Hier will diese Studie einen Informationsbeitrag leisten.

### Vorstellung der Aufsuchenden Behandlungsangebote an den UPK Basel

#### Recovery-orientierte aufsuchende längerfristige Behandlung (Re-ACT)

Die aufsuchende längerfristige Behandlung bietet in den UPK Basel eine Recovery-orientierte und an die individuellen Bedürfnisse angepasste Behandlung für Menschen mit vorheriger hoher Inanspruchnahme von stationären Gesundheitsleistungen an 27 . Das Re-ACT Programm bietet Patient:innen, die die Einschlusskriterien erfüllen (siehe Tab. 1 ) ein zeitlich unbegrenztes Behandlungsangebot an. Das Re-ACT Team umfasste am Ende der ersten Pilotphase 295 % Fachpersonen Pflege Psychiatrie, 30 % Fachärzt:in für Psychiatrie und Psychotherapie und 70 % Sozialarbeiter:innen. Bei der Konzeptionierung des Angebots wurde ein Betreuungsschlüssel von 1:12 bis 1:15 Personen pro FTE angezielt, so dass in etwa 45 bis 60 Patient:innen behandelt werden können.

**Table:** **Tab. 1** Ein- und Ausschlusskriterien der aufsuchenden Versorgungsangebote in den UPK Basel.

Einschlusskriterien	Gemeldet und wohnhaft im Kanton Basel-Stadt
	Alter mind. 18 Jahre
	Hauptdiagnose nach ICD-10 F2x.xx bis F6x.xx; auf Einzelbasis können auch Personen mit anderen Diagnosen aufgenommen werden
	Aktuell in stationärer Behandlung in den UPK Basel
	Mind. drei stationäre Behandlungen und/oder mind. 180 stationäre Behandlungstage in den letzten 2,5 Jahren
Ausschlusskriterien	Keine ausreichenden Deutschkenntnisse
	Ablehnung durch Patient:innen
**Aufsuchende Übergangsbehandlung (ACTTIV)**	
Einschlusskriterien	Gemeldet und wohnhaft im Kanton Basel-Stadt
	Alter mind. 18 Jahre
	Aktuell in stationärer Behandlung in den UPK Basel
	Patient:innen mit Unterstützungsbedarf bezüglich Übergang von stationärer in ambulante Behandlung
Ausschlusskriterien	Keine ausreichenden Deutschkenntnisse
	Ablehnung durch Patient:innen

Re-ACT = Recovery-oriented Assertive Community Treatment; ACTTIV = Assertive Community Treatment – Transitional Intervention; UPK Basel = Universitäre Psychiatrische Kliniken Basel

#### Aufsuchende Übergangsbehandlung (ACTTIV)

In der aufsuchenden Übergangsbehandlung der UPK Basel wird eine Unterstützung des Übergangsprozesses von einer stationären zu einer ambulanten Behandlung angeboten 28 . Sie ist auf drei Monate limitiert. Der inhaltliche Schwerpunkt liegt auf der psychosozialen Behandlung sowie der Wiedereingliederung und Arbeitsaufnahme. Das aufsuchende Team verfügte am Ende der Pilotphase über 405 % Fachpersonen Pflege Psychiatrie, 30 % Fachärzt:in für Psychiatrie und Psychotherapie und 80 % Sozialarbeiter:innen. Auch hier liegt der geplante Betreuungsschlüssel bei 1:12 bis 1:15 Personen pro FTE. Aufgrund von Erfahrungswerten aus anderen Modellprojekten wurde geschätzt, dass im ersten Jahr 250 bis 270 Patient:innen und ab dem zweiten Jahr 370 bis 390 Patient:innen eingeschlossen werden können.

#### Organisation der Programme

Beide aufsuchenden Versorgungsangebote werden für Patient:innen ab 18 Jahren direkt im Anschluss an einen stationären Aufenthalt angeboten (siehe Tab. 1 ).

Teilnehmende Patient:innen können von Montag bis Freitag im Zeitraum von 08:00 bis 18:00 Uhr unterstützt werden. Für die Verbesserung der Erreichbarkeit wurde ein Picket-Telefon innerhalb des Teams eingerichtet. Die kumulative Maximaldauer einzelner Leistungen ist durch die Limitierung im schweizerischen TARMED gegeben (z. B. maximale Leistungsdauer von vier Stunden pro Woche für Fachpersonen Pflege Psychiatrie), kann aber im Rahmen der klinischen Notwendigkeit überschritten werden. Außerhalb dieser Zeiten erfolgt die Versorgung der Patient:innen im Notfall durch die Zentrale Aufnahme der UPK Basel.

Die Behandlungsteams erhalten mindestens einmal pro Monat eine Schulung durch Expert:innen auf dem Gebiet der Gemeindepsychiatrie und der aufsuchenden Behandlung, mindestens einmal wöchentlich Coaching und Fallsupervision und halten mindestens einmal pro Woche eine Teambesprechung ab. Die Mobilität der Teammitglieder wird über die Nutzung von Fahrrädern (E-Bikes) und öffentlichen Verkehrsmitteln sichergestellt. Um die Wege zu verkürzen, arbeiten die Mitarbeitenden wenn möglich in einem zugewiesenen Stadtgebiet.

Beide Angebote sind recoveryorientiert und orientieren sich an dem individuellen Behandlungsbedarf der Patient:innen. Das Erstgespräch findet noch im stationären Rahmen statt. Hierbei wird der Unterstützungsbedarf sowie das bestehende Unterstützungssystem systematisch erfasst.

## Material und Methoden

### Studienablauf und Datenerhebung

Eine Datenerhebung im Jahr 2021 erfolgte mittels halbstrukturierter, leitfadengestützter Fokusgruppeninterviews. Diese wurden mit einem Audioaufnahmegerät aufgezeichnet und im Anschluss mithilfe der Software «MAXQDA 2020.4.1» transkribiert.

Die Einschlusskriterien waren, dass Ärzt:innen mindestens einen Monat lang auf den Abteilungen mit Zuweisungen in die aufsuchende Behandlung gearbeitet haben, um sicherzustellen, dass die Studienteilnehmenden ausreichend Erfahrung mit den Prozessen mitbringen. Alle Teilnehmenden wurden über das Studienziel aufgeklärt und unterschrieben zu Beginn eine Einwilligungserklärung. Die Studie hat im Rahmen der Gesamtevaluation der aufsuchenden Behandlung Basel stattgefunden. Für diese Gesamtevaluation wurde durch die zuständige Ethikkommission ein positives Ethikvotum erteilt (EKNZ 2019-00531).

Soziodemographische Daten der Studienteilnehmenden wurden mittels eines Fragebogens erfasst. Das Alter, Geschlecht, die Berufserfahrung in der psychiatrischen Versorgung wie auch die Erfahrung mit der aufsuchenden Versorgung sowie das Arbeitspensum und der aktuelle Arbeitsort mit dem entsprechenden Behandlungsschwerpunkt wurden erfragt.

Alle Interviews wurden in den Räumen der UPK Basel durchgeführt und jeweils von zwei Personen moderiert (KR und ein:er Assistenzpsycholog:in). Während der Interviews wurde eine Knowledge Map erstellt, um die Diskussionsinhalte strukturiert und zusammenfassend darzustellen 11 . Die Knowledge Map wurde jeweils am Ende der Interviews mit den Teilnehmenden geprüft, um sicherzustellen, dass alle Inhalte richtig verstanden wurden und weitere Aspekte gesammelt und diskutiert werden konnten 11 . Beide Moderator:innen waren den Teilnehmenden unbekannt, ein Kontakt wurde vor den Interviews lediglich über E-Mail hergestellt.

Nach drei Fokusgruppeninterviews wurde eine Wiederholung der Aussagen festgestellt. Von den Teilnehmenden des dritten Interviews wurden nur noch wenige neue Aussagen gesammelt, sodass von einer Datensättigung ausgegangen und die Erhebung abgeschlossen wurde.

### Datenanalyse

Die Datenanalyse erfolgte entsprechend der Inhaltsanalyse nach Mayring 12 . Als Analysetechnik wurde die Zusammenfassung gewählt, die Kategorienbildung erfolgte induktiv. Das Ziel der Analyse ist das Datenmaterial zu reduzieren und ein Kategoriensystem zu erstellen, welches die wesentlichen Inhalte der Interviews abbildet. Die Datensammlung und –analyse erfolgten parallel. Nachdem ein Interview geführt und transkribiert wurde, wurde dieses nach den vorgegebenen Schritten zunächst paraphrasiert und anschliessend generalisiert.

Nachdem alle Interviews geführt wurden, erfolgte die erste sowie die zweite Reduktion der Paraphrasen, sodass bedeutungsgleiche Aussagen auf dem gleichen Abstraktionsniveau zusammengefasst wurden. Auch erfolgte ein Abgleich mit den Knowledge Maps, die während der Interviews erstellt wurden. Nach der zweiten Reduktion folgte eine Zusammenstellung der Aussagen als Kategoriensystem, welches im letzten Schritt noch einmal mit dem Ausgangsmaterial verglichen wurde, um zu überprüfen, ob alle relevanten Themen und Inhalte abgebildet werden. Zur Qualitätssicherung erfolgt die Analyse aller Interviews durch zwei Personen des Forschungsteams (KR und FR).

## Ergebnisse

### Charakterisierung der Teilnehmenden

Drei Fokusgruppeninterviews mit je drei Teilnehmenden wurden durchgeführt. Sie dauerten durchschnittlich 58 Minuten. Alle Ärzt:innen waren zum Zeitpunkt der Interviews an den UPK Basel tätig und in der Zuweisung in aufsuchende Behandlung involviert. Die Teilnehmenden waren durchschnittlich 32,4 Jahre alt (Range: 27–38 Jahre). Drei waren weiblich, sechs männlich. Zwei waren als Oberärzt:innen angestellt, sieben als Assistenzärzt:innen, sechs in Vollzeit und drei mit einem Arbeitspensum von 80%. Zwei der Teilnehmenden wiesen bereits Erfahrungen mit aufsuchender psychiatrischer Behandlung in einer anderen Klinik auf, sieben haben zum ersten Mal in den UPK Kontakt zu einer aufsuchenden Versorgung gehabt.

Die Teilnehmenden waren durchschnittlich seit drei Jahren in der psychiatrischen Versorgung tätig (Range: 0,25–9 Jahre) und durchschnittlich seit 16,1 Monaten in einer Funktion, in der sie in die aufsuchende Behandlung zuweisen konnten (Range: 3–36 Monate). Die Teilnehmenden kamen aus dem gesamten Spektrum der stationären psychiatrischen Angebote an der Klinik für Erwachsene der UPK Basel und repräsentierten Schwerpunktstationen für affektive Störungen, psychotische Störungen, Persönlichkeitsstörungen, Suchterkrankungen und Gerontopsychiatrie.

### Inhaltliche Resultate

Alle Teilnehmenden äusserten sich in den Fokusgruppeninterviews zu ihren Erfahrungen mit der aufsuchenden Behandlung. Mithilfe der Inhaltsanalyse wurden fünf Antwortkategorien aus den Rückmeldungen gebildet. Die Kategorien sind nachfolgend beschrieben und mit Zitaten verdeutlicht.

1. *Anmeldeprozedere:* Der Anmeldeprozess wurde als positiv bewertet. Für die Anmeldung benötigten die Zuweisenden lediglich ein Formular, welches per E-Mail verschickt wurde. Nach der Anmeldung wurde der weitere Prozess von den aufsuchenden Teams übernommen, was als entlastend beschrieben wurde. Nach der Anmeldung fand ein Indikationsgespräch auf der Station statt. Es wurde berichtet, dass die Anmeldung zur aufsuchenden Behandlung recht kurzfristig erfolgen konnte, was sehr geschätzt wurde.
*„Und bisher unbürokratisch und schnell, auch wenn wir zum Teil Patienten haben, die überraschend dann austreten, wenn man nicht so einen langen Vorlauf haben wie man eigentlich gedacht hatte und dann hat es eigentlich trotzdem noch geklappt.“ (Interview 2, Z:35–38)*
2. *Abgrenzung zu bestehenden Versorgungsangeboten in der Region: Die Zuweisenden berichteten, dass es im Kanton Basel-Stadt eine große, relativ unübersichtliche Landschaft von ambulanten Angeboten gibt. Teilnehmende gaben an, dass die aufsuchende Behandlung vorteilhaft ist, weil es ein klinikinternes Angebot ist und dadurch schnell und einfach anzumelden ist.*
*„Und ich glaube dann habe ich das Home Treatment* 1 *manchmal einfach angeboten, weil es für mich leicht anzumelden ist (I:Jaa) Wohnbegleitung läuft über Sozialdienst und auch Spitex auch.“ (Interview 3, Z:220–222)*
  Ein weiterer Grund, der für die Anmeldung des aufsuchenden Angebots benannt wurde, ist, dass in den aufsuchenden Behandlungsteams Ärzt:innen involviert sind.
*„Bei uns ist es glaube ich vor allem eben so, die Komplexität, die entscheidet. Also medizinisch komplexe Patienten mit vielen Medikamenten und so und da weiß da hat man auch einen ärztlichen Ansprechpartner (I:Ja) wo man zum Teil auch sagen kann der sollte direkt involviert auch sein und draufschauen und (I:Mhh) und das macht den Unterschied für uns ja.“ (Interview 2, Z:149–152)*
3. *Patient:innenerfahrungen:* Wenn sie die aufsuchende Behandlung vorstellten, berichteten die zuweisenden Ärzt:innen, dass viele Patient:innen zum Beispiel aufgrund eines hohen Autonomiebedürfnisses zunächst Vorbehalte gegen eine aufsuchende Betreuung im heimischen Umfeld haben. Diese Patient:innen würden meist auch andere ambulante Angebote ablehnen. Auch gab es Berichte von Patient:innen, die sich für ihre Wohnung schämten oder Angst vor Stigmatisierung berichteten, wenn jemand aus dem psychiatrischen Arbeitsbereich zu ihnen nach Hause käme. Die Zuweisenden beschrieben, dass Patient:innen, die schlechte Erfahrungen in der Klinik gemacht haben und auch Patient:innen, die gegen ihren Willen in die Klinik eingewiesen wurden, häufig aufsuchende Behandlungsangebote ablehnten, da sie eine psychiatrische Behandlung nicht fortführen wollten.
*„Ich meine Psychiatrie ist ja auch an sich immer noch ein sehr stigmatisierendes Fach (..) und wo dann auch vielleicht Patienten auch wie du gesagt hast vielleicht auch Ängste haben (..) ehm eben dieses Bedürfnis okay sie sind jetzt hier raus und dann kommt, sie wollen sich komplett zurück ziehen.“ (Interview 1, Z:317–319)*
  Die Zuweisenden berichteten, dass wiederum fast alle Patient:innen, die schon einmal eine aufsuchende Behandlung in Anspruch genommen hatten, diese auch nach dem stationären Aufenthalt wieder wünschten. Dies war für die zuweisenden Ärzt:innen ein gutes Zeichen und bestärkte sie darin, die aufsuchende Behandlung zu empfehlen. Sie berichteten zudem, dass ihrer Erfahrung nach die meisten Patient:innen erst nach Beendigung der aufsuchenden Behandlung wieder stationär einträten, weswegen sie wiederum annehmen würden, dass die Patient:innen, während der aufsuchenden Behandlung gut und wirksam betreut würden.
  *„Aber die Patienten die das Home Treatment auch annehmen, kommen eigentlich auch nie während der Zeit wo sie das Home Treatment haben zurück, ist mir noch nicht aufgefallen* .“ *(Interview 1, Z:464–466)*
  Auch berichteten sie, dass Patient:innen die Flexibilität schätzten, dass die Besuche nicht zwingend bei ihnen zu Hause stattfinden müssen sondern auch Aktivitäten wie Spaziergänge oder Treffen ausserhalb beinhalten können.
  Eine Unsicherheit, die von mehreren Zuweisenden berichtet wurde, besteht darin, dass die Patient:innen bei der aufsuchenden Behandlung über die Inanspruchnahme der Behandlung entscheiden können. Dies bedeutet für Patient:innen, die als non-adhärent eingeschätzt werden, dass sie möglicherweise mit der aufsuchenden Behandlung als alleinige Betreuung unzureichend versorgt sind, da sie eventuell mehrfach die Tür nicht öffnen und die aufsuchende Behandlung dann abgebrochen werden könnte. Andererseits wird von den Interviewten dieser Punkt auch als großer Vorteil beschrieben, da sie erlebt haben, dass die aufsuchende Behandlung von Patient:innen gegenüber anderen Angeboten vorgezogen wird, da die Patient:innen die Kontrolle über die Inanspruchnahme der Termine haben und selbst bestimmen dürfen, wo der Termin stattfindet, ob sie die Tür öffnen und wann die Betreuung beendet wird.
4. *Indikationsstellung der Zuweisenden für die aufsuchende Behandlung:* Die subjektive Begründung für eine Anmeldung wird von den Teilnehmenden heterogen beschrieben. Einerseits, dass ältere Menschen häufig unter Einsamkeit leiden und mit alltäglichen Aufgaben wie dem Haushalt oder administrativen Aufgaben überfordert seien. Bei diesen Patient:innen wurde eine Übergangsbehandlung als aufsuchendes Angebot angemeldet.
*„Naa ich finde es hängt jeweils vom Patienten ab, also es ist ganz bei älteren Patienten ist häufig die Einsamkeit das Thema, das heißt eigentlich das Home Treatment einfach durch den Kontakt (..) ehm bei Sucht Patienten ist wahrscheinlich auch der menschliche Kontakt.“ (Interview 2, Z:189–191)*
  Andererseits wurde beschrieben, dass Patient:innen, die eine Familie oder einen festen Job haben, eine aufsuchende psychiatrische Behandlung eher nicht vorgeschlagen wird, weil man davon ausgeht, dass diese keine weitere ambulante Behandlung benötigen.
*„Also klar Leute, die im Leben stehen mit Beruf und Familie oder so, denen habe ich das nicht angeboten, aber das ist ja eh das sind gar nicht so viele Patienten.“ (Interview 3, Z:185–187)*
  Patient:innen, bei denen die Medikamenteneinnahme unklar ist, werden scheinbar häufiger für eine aufsuchende psychiatrische Behandlung motiviert. Hier sind die Beweggründe unterschiedlich. Einige sagten, dass sie hoffen, dass durch eine aufsuchende Behandlung die Adhärenz gesteigert wird, andere möchten eine Einnahme der Psychopharmaka lediglich sichern. Es gibt jedoch auch Zuweisende, die das Angebot aufgrund der zeitlichen Begrenzung schätzten und nutzten. So wurde von Teilnehmenden, die mit Patient:innen mit Persönlichkeitsstörungen arbeiten, berichtet, dass das Angebot für diese Personen sehr passend sei, da für die kurze Zeit klare Ziele besprochen werden, die innerhalb dieser Zeit erreicht werden können. Auch wurde mehrfach berichtet, dass eine aufsuchende Behandlung solchen Patient:innen angeboten wird, die Hilfe beim Übergang vom stark betreuten stationären Setting zum selbstständigen Setting zuhause benötigen.
*„Und viele (..) haben halt noch kein Übungsumfeld oder sind halt noch nicht so weit, dass sie selbstständig die Sachen anwenden können, die sie gelernt haben auch zum Anspannung reduzieren oder (unv.) die ganzen halt Achtsamkeitsgeschichten und so und ich denke schon, dass da so eine (...) Übergangsbetreuung da schon auch Erinnerungs Reminder mässig so ein bisschen auch (..) wirken kann, auf jeden Fall.“ (Interview 1, Z:249–253)*
5. *Erwartungen und Wünsche der Zuweisenden an die aufsuchende Behandlung:* Die Zuweisenden berichteten, dass sie klare Erwartungen an die aufsuchende Behandlung haben, wenn sie Patient:innen hierzu anmelden. Sie erhoffen sich, dass durch die Betreuung zu Hause Rückfälle vermieden werden können, da Fachpersonen Frühwarnsymptome schneller feststellen können und entsprechende Maßnahmen einleiten können. Auch erhoffen sie sich, dass durch eine aufsuchende Behandlung die Therapie- wie auch die Medikamentenadhärenz der Patient:innen aufrechterhalten oder sogar verstärkt werden.

*„Und gerade auch was die Medis angeht, also ich glaube es ist wichtig, wenn dann wirklich dort teilweise auch abbrechen oder abbrechen wollen (Telefon klingelt) und jemand da ist, der sie auffängt und fragt ‚Hey wie geht es dir damit, hast du irgendwelche Nebenwirkungen oder hast du irgendeine Verbesserung‘ und so weiter.“ (Interview 1, Z:271–274)*

Auch benennen die Zuweisenden Verbesserungsvorschläge bezüglich der aufsuchenden Behandlung. Ein Punkt betrifft den Zeitpunkt der Anmeldung, diese konnte bisher nur während dem stationären Aufenthalt stattfinden, wodurch die Behandlungsplanung und Entlassungsmanagement komplizierter würde. Die zuweisenden Ärzt:innen würden sich hier wünschen, dass auch Patient:innen, die bereits entlassen wurden und in den ambulanten oder teilstationären Status übergegangen sind, noch durch die behandelnden Stationsärzt:innen angemeldet werden könnten.

## Diskussion

An den UPK Basel werden mit der aufsuchenden längerfristigen Behandlung (Re-ACT) und der Übergangsbehandlung (ACTTIV) zwei neue Recovery-orientierte und individualisierte Versorgungsmodelle im Rahmen von Pilotprojekten angeboten. Eine qualitative Befragung der zuweisenden Ärzt:innen zeigt in der vorliegenden Studie, dass die neuen aufsuchenden Behandlungsangebote von den Zuweisenden gut aufgenommen wurden und die Zuweisung bzw. der Anmeldeprozess als unkompliziert beschrieben wird. Die Zuweisenden berichten, dass sie die aufsuchende Behandlung bei ihren Patient:innen als wirksam und rückfallvermindernd erleben.

Wiederholt wurde beschrieben, dass Patient:innen Hemmungen zeigen, ein aufsuchendes psychiatrisches Angebot erstmalig in Anspruch zu nehmen, diese Hemmungen bei vorheriger Inanspruchnahme des Angebotes jedoch reduziert werden können, so dass Patient:innen aufsuchende Folgebehandlungen in der Regel dankend annehmen. Dies spricht für einen guten Beziehungsaufbau und gute Erfahrungen, die Patient:innen während der aufsuchenden Behandlung gemacht haben.

Eine Unsicherheit, die in den Interviews häufiger benannt wurde, ist die Tatsache, dass die Patient:innen die Möglichkeit haben die Tür nicht zu öffnen und die Behandlung damit selbstständig zu beenden. Dies kann bedeuten, dass eine Behandlung möglicherweise nicht oder nicht immer zustande kommt. Andererseits ermöglicht es den Patient:innen und Therapeut:innen, mit der Ambivalenz in Bezug auf die weitere Behandlung konstruktiv zu arbeiten und durch wiederholte Begegnungen eine tragfähige Beziehung in einem Kontext herzustellen, in dem die Patient:innen die Entscheidung bezüglich ihrer (Weiter-)Behandlung treffen. Diese Besonderheit der Autonomie von Patient:innen besteht bei den meisten ambulanten Angeboten und erhöht in der Regel die Akzeptanz des Angebotes.

Bei der Auswahl der Patient:innen spielt auch die Frage, wem eine aufsuchende Versorgung am meisten helfen kann, eine Rolle. Mötteli et al. 13 haben zwei Studien zu schweizerischen aufsuchenden Versorgungsmodellen analysiert und sind zu dem Schluss gekommen, dass es Hinweise gibt, dass Patient:innen mit geringerer Symptomlast und solche, die ganz oder teilweise erwerbstätig sind, am meisten von einer aufsuchenden Behandlung profitieren. In der vorliegenden Befragung der Zuweisenden bildete sich nicht ab, dass diese Patient:innengruppen differenziert zugewiesen wurden. Lediglich wurde berichtet, dass es einige klinische Faktoren gibt, die Zuweisende an eine Indikation für eine aufsuchende Behandlung denken lassen (z. B. drohende Rückfallgefahr, ärztliche Mitbehandlungsnotwendigkeit, komplexe Medikation), dass aber die Auswahl der Patient:innen auch aus praktischen Gründen erfolgt, zum Beispiel aufgrund der vereinfachten Anmeldung im Gegensatz zu anderen Angeboten. Auch wurde beschrieben, dass Patient:innen, die einen Beruf oder Familie haben, eher keine aufsuchenden Behandlungsangebote angeboten werden, oder ältere Patient:innen ein Angebot aufgrund von Einsamkeit erhalten. In den Interviews zeigte sich also, dass sowohl komplex erkrankte Patient:innen mit Komorbiditäten und befürchteter geringer Adhärenz, als auch Patient:innen mit konkretem Unterstützungsbedarf als Zielgruppe gesehen werden. Im Sinne der aufsuchenden Behandlung sollte sowohl der hohe Unterstützungsbedarf der Patienten, als auch die langfristig gute Prognose durch eine niedrigschwellige Intervention, als Indikationen betrachtet werden. Gerade letzteres wurde in den Interviews nur wenig abgebildet. Möglicherweise bedarf es mehr Aufklärung über die Spannbreite der möglichen Indikationen für aufsuchende Angebote.

Des Weiteren zeigte sich in unserer Befragung, dass relativ wenig Wissen über die Angebote der aufsuchenden Versorgung und über die verschiedenen alternativen ambulanten Therapiemöglichkeiten bei den Teilnehmenden besteht. Dies beeinträchtigt eine standardisierte Indikationsstellung für die aufsuchende Behandlung, aber auch für andere ambulante Angebote und führt möglicherweise zu einer suboptimalen Nutzung der verfügbaren Behandlungsressourcen. Diese Interpretation steht im Einklang mit einer Untersuchung von Morant et al. Hierbei wurde von Seiten der Mitarbeitenden von Teams in der aufsuchenden Behandlung ein mangelndes Wissen und Missverständnisse seitens der Zuweisenden sowie negative Erfahrungen bei der Organisation der aufsuchenden Versorgung bemängelt 14 . Eine ähnliche Schwierigkeit wird in der Studie von Schwarz et al. 15 beschrieben. Diese hat sich mit der Implementierung des in Deutschland vertretenen Modells der stationsäquivalenten Behandlung (StäB) beschäftigt. Es wurde geschildert, dass Patient:innen überwiesen werden, die nicht für das ambulante Versorgungsmodell geeignet sind und den stationären Kolleg:innen häufig nicht klar ist, was in der Versorgung tatsächlich passiert und was geleistet werden kann. Eine weitere Studie von Lang et al. 16 hat sich mit den Hindernissen der StäB beschäftigt. Auch dort wurde gezeigt, dass beispielsweise die Schwere der Erkrankung, aber auch Einfluss von Angehörigen oder die Wohnsituation selbst zu einem Abbruch der Behandlung führen kann. Die Autor:innen führten dabei an, dass auch eine ausführliche Abklärung der Rahmenbedingungen einen Abbruch der Behandlung nicht verhindern konnte, da sich Schwierigkeiten häufig erst im Alltag zeigten 16 . Dies deckt sich mit den Erfahrungen aus den beiden Angeboten der UPK. Gleichzeitig zeigte die Studie von Lang et al. auch, dass die starren Rahmenbedingungen der StäB (beispielsweise die täglichen Kontakte) ein Hindernis sein konnten und auch zum Abbruch der Behandlung führten 16 . Die Flexibilität der Rahmenbedingungen wurde in den beiden Projekten der UPK mehrfach positiv erwähnt und scheint einen wichtigen Bestandteil bei der Akzeptanz des Angebots zu spielen.

Die grössten Unterschiede zu dem aufsuchenden Angebot der UPK im Vergleich zu anderen aufsuchenden Angeboten sind einerseits die strukturellen Kriterien (Verfügbarkeit 24h etc.) und andererseits die Grundidee des Angebots. Die StäB soll als eine Alternative zum stationären Aufenthalt angeboten werden 17 und erfolgt daher in der Regel nicht nach einem stationären Aufenthalt, wie es beim Angebot der UPK der Fall ist, sondern wird anstelle des Aufenthalts angeboten. Zudem wird bei der StäB eine 24h Erreichbarkeit (ein aus Nutzer:innenperspektive wichtiger Faktor 18 19 ) sowie ein täglicher persönlicher Kontakt gewährleistet 17 . Dies ist in dem Konzept der Angebote Re-ACT und ACTTIV nicht vorgesehen. Andere Angebote in der Schweiz haben ebenfalls unterschiedliche Rahmenbedingungen und Kriterien 20 21 . Die Tatsache, dass ausschliesslich stationäre Patient:innen zugewiesen werden können, wurde in den Befragungen unterschiedlich bewertet. Einerseits wurde dies als Hindernis beschrieben, da Patient:innen nicht entlassen werden können, um dann beispielsweise eine Woche später ein Indikationsgespräch zu erhalten. Dies könnte eine zeitliche Entlastung bedeuten, jedoch auch dazu führen, dass keine nahtlose Weiterbehandlung gewährleistet ist. Andererseits wurde beschrieben, dass Patient:innen durch den vorherigen stationären Aufenthalt in manchen Fällen geprägt wurden. War für Patient:innen der stationäre Indexaufenthalt in der Klinik schon positiv (vs. negativ), haben sie möglicherweise mehr (vs. weniger) Vertrauen in eine aufsuchende Behandlung, die von der gleichen Klinik angeboten wird.

### Stärken und Limitationen

Die Stichprobengrösse der zuweisenden Ärzt:innen ist moderat, jedoch konnte mit den Teilnehmenden an der aktuellen Untersuchung eine Datensättigung erreicht werden, so dass sie als ausreichend erscheint. Auch wurden die Gütekriterien eines qualitativen Verfahrens eingehalten 22 . Die Befragungen beziehen sich auf zwei spezifische aufsuchende Angebote innerhalb zweier Pilotprojekte einer großen schweizerischen Universitätsklinik und fokussierten besonders auf die Perspektive der Zuweisenden. Damit liefern die Ergebnisse wichtige Informationen, die für die Implementierung dieser und ähnlicher aufsuchender Dienste von Nutzen sein können. Ihre Generalisierbarkeit auf andere aufsuchende Angebote und Versorgungsregionen könnte jedoch eingeschränkt sein, da Angebote oft stark variieren 23 . Zudem wurde in dieser Arbeit lediglich die Sichtweise der zuweisenden Ärzt:innen betrachtet. Der Einbezug weiterer Perspektiven, namentlich der anderer Mitarbeiter:innen, der Patient:innen (vgl. auch 18 19 ) und der Angehörigen in weiteren Erhebungen würde helfen, ein umfassenderes Bild darzustellen. Auch ist es wichtig die Effekte der aufsuchenden Behandlung quantitativ zu evaluieren 24 ; erste positive Hinweise für die hier vorgestellten Programme konnten zeigen, dass die aufsuchende Behandlung mit weniger stationären Neuaufnahmen und Behandlungstagen im 6-monatigen Beobachtungszeitraum assoziiert ist 25 .

## Fazit für die Praxis

- Die aufsuchenden Behandlungsangebote sowie der interne Prozess zur Anmeldung haben sich aus Sicht der Zuweisenden bewährt und wurden als positiv bewertet. Eine noch flexiblere Gestaltung des Zuweisungsprozesses inkl. einer Aufnahme ausserhalb der stationären Behandlung ist gewünscht.
- Bei Behandler:innen scheint eine unzureichende Übersicht über die lokale Versorgunglandschaft zu bestehen. Eine verbesserte Übersicht könnte zu einer gezielteren Auswahl aufsuchender Angebote führen.
- Wenn Patient:innen Vorbehalte gegen eine aufsuchende Behandlung berichten (z. B. Angst vor Stigmatisierung bei Hausbesuch), können diese laut der Zuweisenden durch eine Erstinanspruchnahme einer aufsuchenden Behandlung meist korrigiert werden. Eine motivierende Gesprächsführung der Zuweisenden mit dem Vorschlag, die aufsuchenden Behandlungsangebote ggf. einmal auszuprobieren und bei Bedarf wieder abzubrechen oder das aufsuchende Behandlungsteam trotz Vorbehalten kennenzulernen, scheint angezeigt, um die Hürde bei den Patient:innen für eine erstmalige Inanspruchnahme zu verringern.

## Footnotes

1
*Zum Zeitpunkt der Interviews wurde das Pilotprojekt als Home Treatment bezeichnet, weswegen diese Bezeichnung in den Interviews genutzt wird. Inhaltlich gab es jedoch keine Veränderung.*
